# Education Research: Explaining Neurophobia in Medical Students: A Comparison With Math Anxiety

**DOI:** 10.1212/NE9.0000000000200340

**Published:** 2026-07-13

**Authors:** Pearce J. Korb, Ian McGuinness, Trevor Hawkins, Janet Corral

**Affiliations:** 1 Department of Neurology at Virginia Commonwealth University in Richmond;; 2 Neurology Consultants of Huntsville, PC in Huntsville, AL;; 3 Department of Neurology at the University of Colorado School of Medicine, Aurora; and; 4 CEO of Enlighten Strategies Corporation, Reno, NV.

## Abstract

**Background and Objectives:**

Neurophobia, or the fear of the study and practice of neurology or neuroscience, plagues learners and practitioners of all types. Neurophobia is a global phenomenon and adversely affects medical education, the field of neurology, and ultimately patient care. Despite this, there is still much to be understood. It is reasonable to compare this relatively undefined phenomenon in medical education to lack of confidence or anxiety in other areas of education that are more well-understood. One of the best studied is the fear of mathematics, often referred to as math anxiety (MA). MA has 3 major causal categories relating to the learning environment, the material itself, and characteristics of the learner. We aim to have a deeper understanding of the causes of neurophobia for medical students while comparing this with MA.

**Methods:**

This study used constructivist grounded theory, informed by MA, to understand the causes of neurophobia for medical students. Four groups of 6 to 8 medical students from each year of the curriculum were recruited purposively and engaged in semistructured focus group discussions guided by sensitizing concepts of MA until saturation, or thematic adequacy was reached.

**Results:**

We identify 5 major themes and 18 subthemes related to neurophobia. Three of these themes are comparable with MA and relate to the Environment, the Material, and traits of the Learner. The Environment includes influential experiences during and before medical school. The Material includes the influences of both the content and curriculum. The Learner includes factors of student self-concept and confidence. One emerging theme includes the “Black Box” phenomenon, or the inscrutability of neurology, in both the opacity of the material and isolation throughout the curriculum. Another theme, we call Career Perspective, is how the student's perceived relevance and utility of the subject profoundly effects neurophobia.

**Discussion:**

We propose a new model for neurophobia partially informed by MA. It acknowledges the effect of each individual theme and their relationship with each other. We suggest this model has important implications for more effective interventions aimed at addressing neurophobia in medical students and directing future research.

## Introduction

Neurophobia, or the fear of the study and practice of neurology and neural sciences, plagues learners and health practitioners of all types and adversely affects patient care.^1-3^ Coined in 1994, neurophobia is the perception that neurology is the most difficult subject of all medical specialties and is well-established as a global phenomenon.^4-14^ Fear, or perhaps better and more recently characterized as anxiety,^13^ for this field is particularly concerning because neurologic disease represents a large proportion of worldwide disease burden. This underscores a high demand for neurologists; however, neurophobia and related curricular choices influence the number of medical students entering neurology and potentially limit the supply.^15,16^ Since these patients are most often first managed by non-neurologists, there is also a pressing and growing need for non-neurologists comfortable practicing core neurology.^6,9,11,17-21^ Neurophobia, and the resulting gap in expertise, leads to detrimental practice changes, including excessive referral rates, increased wait times, and decreased access, ultimately adversely affecting patient care.^2,3^

Despite its ubiquity and harm, there is still an evolving definition and emerging but limited in-depth causal analysis. Therefore, it has been difficult to develop consistent conceptual and explicit theoretical frameworks for neurophobia. Consequently, there is limited progress toward a solution for curricular leaders and teachers. It is time to look to other fields for guidance.

### Current Data on Neurophobia Factors

Across several studies examining possible reasons for neurophobia, learners identify some consistent factors: vast amounts of requisite knowledge in neuroanatomy and neuroscience, the complexity and number of diagnoses, the complexity of the neurologic examination, insufficient exposure to patients, poor teaching, and negative perception of the field before school.^4-6,8-12,22^ There is a lack of data on how or why these elements induce neurophobia.^14^

One study elaborates on 2 important phenomena. They show neurophobia often predates any formal curriculum, concluding that preconceived negative perceptions about neurology have a significant influence.^22^ Second, they also show the perception of neurology changes throughout the curriculum with confidence highest after any neurology curriculum, but rapidly wearing off.

There remain questions about the complex relationship of the learner to the field. How much does the material or the curricular design contribute? How about self-perception or perceived ability? A deeper level of understanding would be beneficial in formulating plans to address neurophobia.

### Summary of Limited Pedagogical Interventions

The extant literature on interventions for neurophobia focuses on the effect of individual learning formats implemented during specific points in the overall curriculum such as problem-based learning,^23,24^ team-based learning,^25,26^ and others.^27-32^ Fewer examine the effect of macro curricular considerations, such as a more longitudinal clinical curriucula,^7^ and early mentoring and clinical experiences.^31,32^ Some recent reviews and viewpoints discuss the state of the literature and future directions including: the lack of high-quality evidence for effective interventions,^33^ the need to underpin the studies with an existing learning theory or conceptual framework,^34^ calling for standard measurement tools, more study into how to combat neurophobia and generate confidence and interest in neurology.^14^

### Math Anxiety

To build a more explicit conceptual framework or model for neurophobia, it is reasonable to compare it to lack of confidence in other subjects. Forty years before Dr. Jozefowicz popularized the term “neurophobia,” a Catholic nun teaching in Spalding, Nebraska, Sister Mary Fides Gough O.P., coined the term “mathemaphobia” to describe what is now known as math anxiety (MA).^35^ Both are highly prevalent among adult learners; in the United States, 25%–80% of college students have math anxiety,^36^ and nearly 50% of medical students endorse neurophobia.^37^

Studied since the 1970s, MA is commonly defined as a “feeling of tension, apprehension, or fear that interferes with math performance” (pg. 181).^38^ Math anxiety has strong negative correlations with confidence, motivation, and satisfaction with the subject matter.^39,40^ It is also associated with lower levels of achievement in math courses and real-world tasks. It results in avoidance of further study and is therefore a self-perpetuating effect.^39,40^

As outlined in Table 1, some of the causal factors associated with MA are divided into 3 categories: Dispositional, Situational, and Environmental.^41^ Dispositional factors include traits inherent to the student. Situational factors include those external to the student, but relate to the subject itself, for example, the complexity of trigonometry.^42^ It can also refer to the curriculum or how the subject is taught. Environmental factors are related to external influences from the past or surroundings, for example, prior negative experiences with teachers.^43^

**Table 1:** Math Anxiety

Causal factors for math anxiety		
Category	Description	Example
Dispositional	Traits inherent to the student	Low self-esteem or self-concept; having low math ability; poor study skills; negative attitude toward math
Situational	Factors related to the subject or curriculum	Complexity of math processes; teacher centered learning approaches; poorly aligned assessments
Environmental	Prior influences from external sources	Negative experiences in the classroom; low expectations from parents, teachers, or mentors

### Purpose

The intent of this study is to have a deeper understanding of the causes of neurophobia for medical students and compare this with math anxiety.

### Research Questions

1. To what extent are the causal factors of neurophobia comparable with those of math anxiety (dispositional, situational, and environmental)?
2. What are the unique features of these causal factors that contribute to, or protect against, neurophobia?

## Methods

### Methodology

We approached the study with a constructivist worldview, an education philosophy in which learners construct their knowledge and ideas, and our belief that neurophobia is, in part, a social construct. We used constructivist grounded theory, an explanatory qualitative research methodology that includes induction (“an open mind”) to inform the study design. This approach was selected because it is well suited to study potentially complex social phenomena, subjective learning experiences, and concept development while acknowledging our role as researchers in its generation and other sensitizing concepts. It was theory informed, and therefore not purely inductive, by the literature review of neurophobia and the phenomenon of MA serving as sensitizing concepts. We used the established characteristics of MA to provide a point of reference for our study into neurophobia.

### Participants

**Figure 1:**
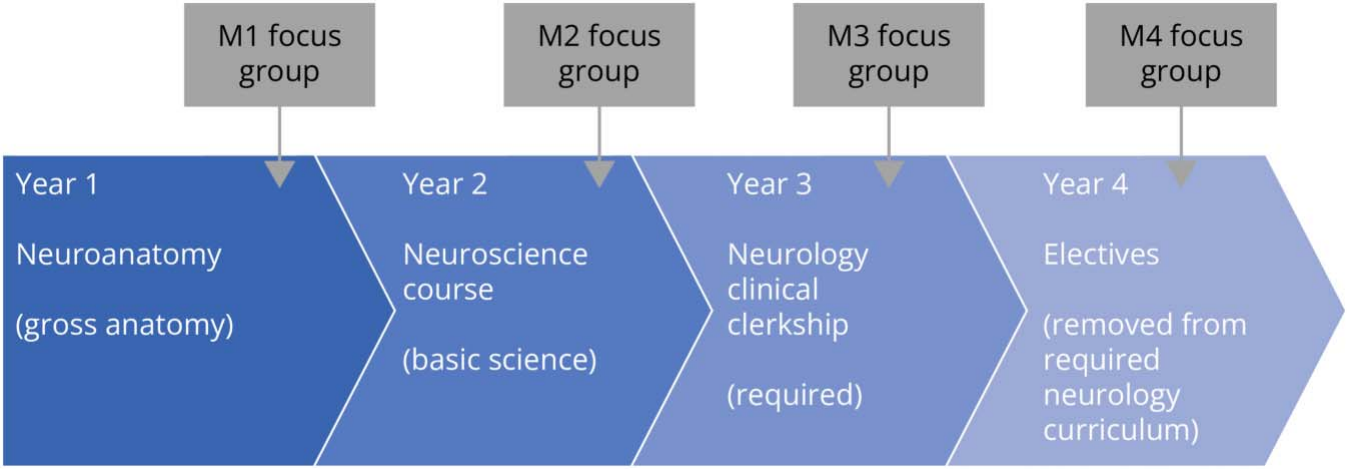
Timeline of Neurology Curriculum and Composition of Focus Groups

The study included medical students at the University of Colorado School of Medicine (CUSOM). At the time of the study (2019), the program duration was 4 years with 2 years of preclinical curriculum followed by 2 years of clinical clerkships. In terms of the neurology curriculum, neuroanatomy was part of a gross anatomy course in the first year, a neuroscience course in the fall of the second year, and a required clinical clerkship in the third year. Electives in research and further clinical rotations were available in the fourth year (Figure 1). The curriculum was supported by a large Department of Neurology with over 40 full-time clinical faculty. Based on survey results of medical students, local confidence in the study of neurology and neuroscience was low and comparable with the literature review.

### Recruitment

We used purposive, homogenous sampling by inviting students from each class for a common educational context. The invitations to participate in the voluntary study were generated randomly and sent via email in waves until the ideal number of participants in each group was reached. The subject of the focus group was presented as a general discussion on curriculum, without mentioning neurology or neurophobia, to allow for exploratory analysis and have groups of students with potentially different perspectives on neurology.

### Data Collection

There were 4 focus groups consisting of 6 to 8 students from each of the following cohorts: Year one students (M1) before any neurology curriculum neurology exposure, Year 2 students (M2) after the neuroscience course, Year 3 students (M3) after the neurology clinical clerkship, and Year 4 (M4) students about to graduate and removed from the required neurology curriculum (Figure 1). The discussions were scheduled during weekdays, during work/school hours, for 2 hours and moderated by T. Hawkins and I. McGuinness, both neurology trainees at the time of study.

Focus group questions followed a semistructured discussion guide (eAppendix 1) developed and reviewed by experts in qualitative methodology and medical education research. Questions focused on sensitizing concepts and causal factors from prior studies on both neurophobia and MA. For example, the core questions were framed after the 3 categories of MA (Table 1, eAppendix 1). The moderators were trained in several key practices for focus group discussions: to follow-up questions on the sensitizing concepts, be open to discussion outside the sensitizing concept, gently probe, explore conflicting viewpoints, and note nonverbal communication. Questions evolved based on concepts generated throughout data collection.

The discussion groups were conducted in-person, during the day, and were moderated by investigators who had no administrative relationship with the students. The sessions were audio recorded and transcribed verbatim, with notes from the moderator added. In between sessions, the researchers met to discuss the data and adjusted the questions in the discussion guide for subsequent discussions. There were checks for saturation, or thematic adequacy, and we did not identify new themes related to the study questions after the last planned session therefore stopped data collection at that point.^44^

### Analysis and Reflexivity

The transcripts were first read several times to get a general sense of the results. The reviewers, I. McGuinness, T. Hawkins, and P. J. Korb, then open-coded the information and identified initial themes or sensitizing concepts. Triangulation, by validating prior findings and analysis with other participants, was conducted throughout the study. The research team met frequently to review the transcripts and codes until final themes and consensus were obtained. All investigators kept a diary throughout the study to explore and document their relationships to the data, topic, and participants to encourage reflexivity. All the researchers involved in data collection and interpretation have chosen careers in neurology, do not suffer from neurophobia, but conversely have a passion for the field. In addition, all participated in the literature review and have a knowledge of neurophobia and MA. An audit trail was kept throughout. Final themes and codes were informed a priori by the dispositional, situational, and environmental causal factors for neurophobia. The final analysis was conducted to explain all potential causal mechanisms for neurophobia and a conceptual framework and model was generated from this work.

### Standard Protocol Approvals, Registrations, and Patient Consents

The study was approved by the local ethical and investigational review board, and informed consent was obtained from all participants before data collection.

### Data Availability

Data not provided in the article because of space limitations may be shared (anonymized) at the request of any qualified investigator for purposes of replicating procedures and results.

## Results

We conducted 4 focus groups: one for each class of medical students (M1-4). Thirty students participated including 15 women and 15 men. We identified 5 major organizing themes and 18 subthemes (Table 2). Selected quotes are presented in Table 3, while the more extensive collection is listed in eTable 1. Within the text, we present individual quotes with quotation marks and italicize commonly used terms used by participants.

**Table 2:** List of Major Themes in Neurophobia for Medical Students

**Theme 1. The Environment—there are significant external influences on neurophobia**	
The external influences can be from multiple sources resulting in bias of the field, the professionals, or the study of neurology	
Over time environmental influences on neurophobia occur at different points and can persist	
**Theme 2. The Material—the subject matter and how it is taught affects neurophobia**	
The following factors related to the study of neurology significantly affects neurophobia:	
Subject matter	Elements inherent to the study and practice of neurology
The Diseases & Pathology	The clinical conditions associated with neurology
Abstraction	The abstract or complicated nature of the material
Amount of content	The large quantity of material
Curriculum	How the material is delivered
Clinical relevance	The amount (lack) of clinical context in the material
Teaching	The quality of teaching or effect of individual teachers
Over time material factors change with each milestone but gains in confidence wear off	
**Theme 3. The Learner—the skills and attitudes of the student affect neurophobia**	
The following inherent factors of the learner influence neurophobia:	
Self-concept	Self-reflection of the ability to practice or study neurology
Skills	Specific academic skills are protective against neurophobia
Reciprocal	The extent to which performance affects anxiety and vice versa
Over time learner factors of neurophobia change based on environmental and material factors	
**Theme 4. The Black Box—wide-spread isolation of neurology worsens neurophobia**	
The following forms of isolation of neurology from itself and the rest of the curriculum:	
Environmental Black Box	The external influences that create a sense of isolation
Material Black Box	Subject matter or curriculum that separates neurology
**Theme 5. Career Perspective—the more learners relate their confidence to a career commitment in neurology increases neurophobia in multiple ways**	
Increases environmental-related neurophobia	
Increases material-related neurophobia	
Increases learner-related neurophobia	
Career bias over time	

**Table 3:** Selection of Representative Quotes

Theme/subtheme	Representative quote
Environment	
Personal experiences with neurologic disease	I have a family member who has a neurologic disease. […] I've just kind of seen how bad it can get and more, […] That's where my hesitation comes in. […] (#M1.1)
Society, media, and popular cultural influence	You hear people say, “It's not rocket science, it's not brain surgery.” People hold it on this pedestal of the most difficult […] these preconceived notions before you even learn how complicated the body is, especially neurology. (#M2.3)
Material	
The abstract material	In anatomy the structure looks like the function it should do. Like, you see your heart and you're like “Oh that's a pump.” […] that looks like what it should do. But when you look at a brain? [group laughter]. (#M2.4)
The amount of material	It is like trying to grab a big pile of laundry and every time I try to grab one more, something else falls out (#M4.6)
The lack of clinical context	I think for me the second-year (preclinical) curriculum made it scarier than it actually was. […] But when I was actually in the clinic (clerkship) with my preceptor, it was more about like general localization and not so much like the minutiae. (#M3.3)
The teaching	[…] I was literally like, “Why are they here talking about this?” […] they just spent 40 min talking about their own research and it's like so out of the scope of like what we need to know right now. (#M2.6)
The Learner	It, probably a lot of the time, stems from feelings of inadequacy, feelings of not being intelligent enough, or that the field itself is just so much more challenging than any of these other ones. (#M1.2)
The Black Box	The brain is like the black box where electricity goes in and then electricity goes out. And no one really knows what happens up there. (#M3.5)
	As a third-year student I feel like on no other rotation did we talk about neurologic problems. So, before this clerkship [neurology] I was genuinely worried about it because I hadn't been talking about any of these conditions over the past 12–15 mo (#M3.1)
Career Perspective	There is no fear in my mind. There is no fear of failure. There is fear of commitment. I think it's more like, do you want to do this for the next 20–30 y of your life. (#M1.1)
For a more expanded selection of representative quotes, please see eTable 1 in the supplemental material	

Most of the participants endorsed some anxiety or fear consistent with neurophobia. This was not quantified, but there was at least unanimous appreciation of the concept. The students were able to discuss specific drivers, supporting examples, and engaged in active discussions. Themes generated came from consensus, but we also noted important contradictions. Not all the students' experiences had the same impact. For example, the complexity of neuroscience in the preclinical courses usually caused anxiety but was a source of curious inspiration for a minority of the students.

Three of the major themes for neurophobia correlated to math anxiety and included influences from the Environment, the Material, and the Learner (note: these terms were chosen for clarity by the research team to represent the terms used in MA literature: environmental, situational, and dispositional, respectively). There were also 2 emergent, specific themes for neurophobia. One included the effect of the isolation of neurology and neuroscience teaching and material, or what we called the “Black Box” phenomenon. The other was the insidious effect of perceived meaning, utility, and relevance learners assigned to neurology, which we called Career Perspective. We described these themes and subthemes in more detail below.

### Environmental Influences on Neurophobia

Experiences with the field, people, or study of neurology influenced confidence in neurology. We distinguished this concept from the commonly used medical educational term, “learning environment,” a more encompassing term for all elements in learning settings. Our use of “environmental factors” referred to experiences outside of the explicit curriculum. These experiences could have occurred before or during medical school. They had the potential to engender passion for the field, but more often worsened neurophobia.

Experiences involving patients and family members with a grim prognosis, that is, neurodegenerative disease, had a particularly deleterious effect. Generally, impressions of neurology providers themselves were good, even in comparison with other common fields. Detrimental influences also came from popular culture and media and from non-neurology healthcare providers.

### Material Influences on Neurophobia

Material referred to the elements of both what was taught (subject matter) and how it was taught (curriculum), each having independent effects on neurophobia.

#### The Subject Matter

For many of the students, “the subject matter is the overarching fear factor” (#M2.2). There are several reasons why the subject matter effects were independent of how well it was taught. Some of the most frequent themes were the (1) pathology and diseases in neurology, the (2) abstract nature of the material, and the (3) vast amount of content.

One of the most frequent themes was the diseases and pathology of neurology. The perception of neurology dealing with diseases with poor prognosis, poor outcomes, and limited therapeutic options increased neurophobia. Another related concern was the *high stakes* of brain diseases. Students expressed anxiety dealing with pathologies that are a threat to their own *sense of self.* In addition, the *abstract* nature of the material generally contributed to neurophobia. They often referred to neuroanatomy with mental visualization of the structure-function relationships being more difficult than in other core subjects, for example, cardiology.

Not only was the material abstract, but it was vast in scope and overwhelming. This vast content existed in both the neuroscience and neurology curriculum and included the multiplicity of the terminology, neuroanatomy, and the neurology examination. One student eloquently recalled their experience in neuroscience as “trying to grab a big pile of laundry and every time I try to grab one more, something else falls out” (#M4.6).

#### The Curriculum

The delivery of the material also affected neurophobia. Students generally thought the neuroscience unit was taught well, but there was a lack of clinical context presented with the basic science material. This increased the struggle with abstract material and worsened neurophobia before the clinical clerkship. The quality of teaching affected neurophobia bidirectionally. Factors for neurophobia included issues with unrestrained scope, lack of focus, and content coordination with other parts of the course. Teaching strategies that alleviated neurophobia included: giving expectations of success, having a positive attitude towards the students, innovative visual formats, and scaffolding and clear organization of the material.

### Learner Influences on Neurophobia

Having a poor self-concept about one's general academic abilities worsened neurophobia. These students' self-concept was highly influenced by both the ongoing Environmental and the Material factors. This made it difficult to separate the Learner from external factors. Examples of Environment triggers included emotionally harmful prior experiences in neurology or neuroscience. Conversely, for some students, prior academic struggles evolved into academic skills, such as improved study habits or resolve, which were protective against neurophobia. Material factors that heavily affected the learners' self-concept and led to *imposter syndrome* were the high stakes of the material, its abstract nature, and general fear of the unknown.

### The Black Box

Neurology and neuroscience were considered enigmatic with a sense of isolation or “estrangement” throughout and beyond the curriculum. *The brain is like the black box where electricity goes in and then electricity goes out. And no one really knows what happens up there* (#M3.5).

This sense of “otherness” and mystery was apparent in 2 distinct ways: the Material and the Environment. As for the Material, there was an expression of the “brain” itself as a *black box,* referring to the comparative complexity of neuroanatomy, neurophysiology and related pathology as a subject matter issue, but there is also curricular isolation. There was a lack of teaching neuroscience outside the neuroscience course, and neurology outside of the single 4-week neurology clerkship.

This isolation of neurology was also related to the broader Environment. The black box phenomenon also occurred as unintentional, hidden elements within and outside the neurology curriculum entirely. For example, before medical school or in popular culture, neurology was seen as elevated or “propped up on this pedestal” (*#M1.6*) which could negatively affect the perception of the field. In the clinical curriculum, there was isolation of neurology from other forms of clinical medicine. Their supervisors in other fields also expressed neurology as a *black box,* as evidenced by the frequent need for consultation on non-neurology rotations. These collective *black box* features of opacity and nonintegration uniformly worsened neurophobia.

### Career Perspective

The theme of Career Perspective referred to how learners spontaneously and consistently weigh their confidence in neurology to its potential impact on their professional future. Any neurophobia was amplified when students envisioned neurology as a significant portion of their careers. Conversely, when students presumed neurology would not be a substantial part of their career, the importance of any neurophobia, and neurologic education in general, was diminished. This variable perception would raise or lower the stakes of neurophobia through the other aforementioned factors, for example, the engagement of the material (Material), interpretation of external influence (Environment), and self-concept (Learner).

## Discussion

**Figure 2:**
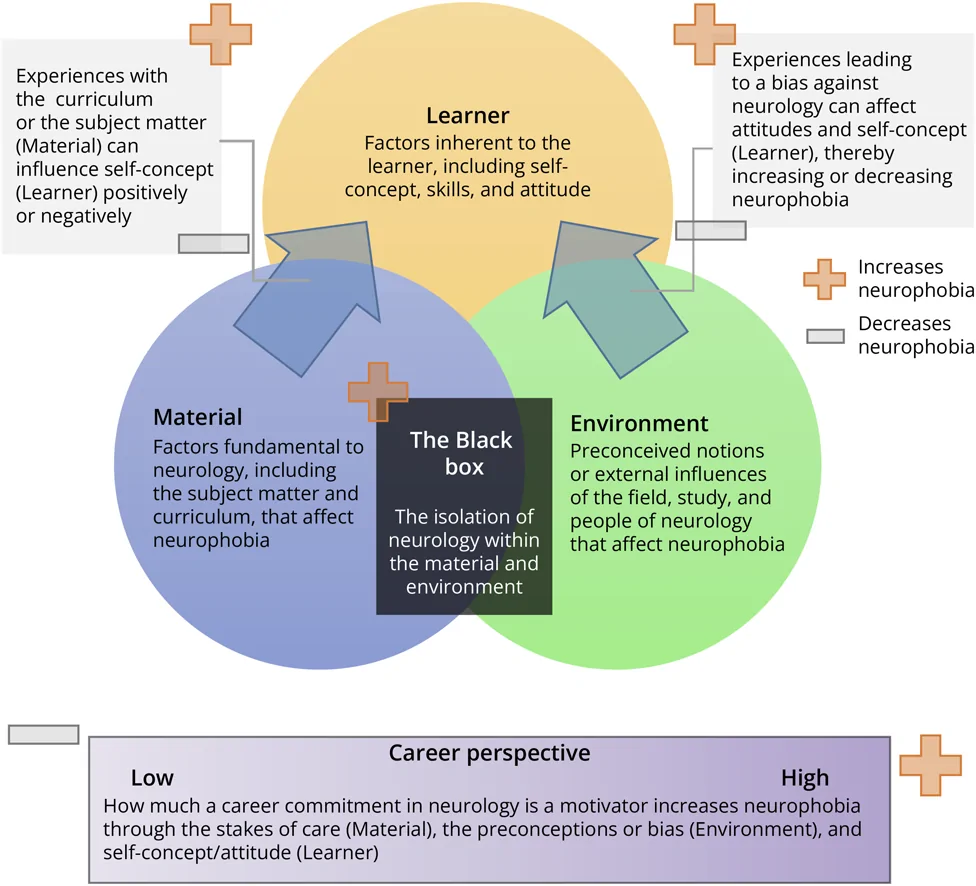
Neurophobia Model The factors could be categorized similarly to math anxiety (MA). Major factor categories included the Learner, Material, and Environment, which are analogous to the Dispositional, Situational, and Environmental Factors in MA, respectively. There is overlap in the categories with bidirectional effects on neurophobia. Special attention is brought to the “Black Box” of neurology. In addition, the level of Career Perspective of the learner affected all categories.

The aim of this study is to have a deeper understanding of the causes of neurophobia for medical students and compare this with math anxiety. There are 5 major themes or factors for neurophobia including: 1. environmental factors, 2. materials factors, 3. learner factors, 4. the Black Box phenomenon, and the 5. career perspective factor. After analysis of the 5 major themes with 18 subthemes, we offer a model to better understand how these factors affect neurophobia and offer potential “healing” strategies (Figure 2).

This model has several important dimensions. First, there are the 3 central themes for neurophobia mirroring the ones for MA. The names are changed to be more self-evident; they are Environment, Material, and Learner factors.

For each of the 3 factor categories there is overlap, so they are represented as a Venn diagram (Figure 2). There is also a general consequential direction for the Learner, represented by arrows. Each has factors with both potential aggravating or alleviating effects on neurophobia, represented by “+” or “−” signs (with “+” representing something that worsens neurophobia and “−” as alleviating). The Black Box phenomenon has both Environmental and Material influences so overlaps both. Finally, there is one theme, Career Perspective, presented as an overarching factor affecting all other categories.

Several established factors of neurophobia are confirmed as important contributors to neurophobia, including (1) the vast and overwhelming amount of content, (2) the nature of the diseases,^45^ and (3) the effect of prior experiences.^22^ There are some important elaborations, including the ambiguity and multiplicity of the terminology, lack of integration of the basic and clinical concepts, abstract nature of the material, unclear diagnoses with complicated evaluations and treatments, and role of non-neurologists in perpetuating a negative bias.

There is very little in the neurophobia literature about the inherent factors of the Learner, limited to demographic predictors or career interest effects on neurophobia.^37^ Our data focus on self-concept which is not yet fully addressed in the literature. However, there is some emerging interest in designing neuroscience courses with learner focused frameworks such as self-determination theory.^46^

There are major similarities in causes of neurophobia and MA; however, there are also some important differences.

Neurophobia is influenced by several sources external to the learner, and MA has comparable Environmental influences: (1) prior exposures to the subject in the classroom and real world, (2) the social interaction with others experienced in the subject,^47^ and (3) a similarly dubious cultural reputation as being difficult.^48^ Awareness of these factors outside the direct curriculum could inform stakeholders and curriculum designers, as they have for MA.

In addition, material factors are important to both neurophobia and MA. Despite obvious differences in the subject matter between math and neurology, the nature and complexity of both subjects relate to anxiety in each. Recognizing that the subject is interesting, but also complex, could inform strategies such as scaffolding, explicit expectation setting, and labeling of essential and nonessential material.

There are learner factors in both neurophobia and MA. There is direct evidence that poor performance is linked to subsequent MA^40^; however, there is no such link yet proven in neurophobia. This would be harder to prove as the MA studies can measure test anxiety and performance in a more sterile classroom environment,^38^ whereas assessments in neurology are more work-based, qualitative, and complicated. These focus groups express the importance of self-concept on neurophobia, and there is evidence that self-concept is more meaningful than performance in MA anyway,^43^ thus making this comparison valid. Curriculum designers should consider learner centered concepts such as self-efficacy and self-determination theories in their designs.

Two themes do not clearly correspond to MA and do not appear extensively in the neurophobia literature.

As presented earlier, one student describes the brain as a “black box where electricity goes in and then electricity goes out.” This reflects the complicated nature of the material but could also be an analogy for the widespread isolation and relative inaccessibility of the neurology curriculum; therefore, we depict it as overlapping the major categories of Environment and the Material in the model (Figure 2). One key discovery from these data is the isolation being reinforced throughout the career of a student, including before medical school. This also occurs during a critical time in the clinical clerkships, as the professional teams perpetuate neurology as “different” from all others with their actions. This comprehensive feeling of isolation or the black box of neurology has a profound, insidious effect on neurophobia. This has potential profound implications for educators as addressing neurophobia need to look outside the bounds of the neurology and neuroscience curriculum.

The other unique, surprising, and important finding is the effect of Career Perspective on neurophobia. How much a student perceives, accurately, or not, neurology will be a part of their career drives the importance they assign to neurophobia and neurologic education. It is natural and expected for medical students to use their experience in the curriculum to influence their professional identity and career choice.^49^ However, this involuntary emphasis on neurology mainly as a career consideration interferes with motivation in attaining general competency in neurology. This is concerning because most neurologic problems present to non-neurologists.^6,9,11^ We need more neurologists, but we also need more non-neurologists practicing core neurology.

How should educators address this dual challenge? First, students need to have an accurate understanding of how neurology will be a part of their practice, even if they do not become neurologists. Second, educators must design the curriculum to reflect both goals of training non-neurologists and recruiting more neurologists.

The purposive and homogenous nature of the sampling method did specifically select for students who either identify with or without neurophobia. Without specifically looking for contrary opinions, this potentially limits a more credible discussion. Ultimately, the sampling method is reliant on volunteers and thus might affect transferability. This study is also limited to a single site, potentially affecting its credibility. Although the number of sessions and students was predetermined based on best practices, saturation, or thematic adequacy, had to be determined during data collection. Focus groups, opposed to interviews, can encourage dynamic interactions to explain the phenomenon and may provide richer details. However, they may create an environment in which sharing more personal or sensitive information is difficult and may potentially be missed. With only a single focus group moderator for each session, a lack of a second observer to collect all nonverbal information may limit the data and interpretation. Constructivist grounded theory informed by a mixture of predetermined codes based on the sensitizing concept of MA and open coding may have created a bias to fit the model.

We feel strongly that interventions to improve neurophobia should be based on a better understanding of the phenomenon. Even recent calls to fight neurophobia suggest a response with modern techniques but without a clear foundation of what neurophobia is.^30^ To reduce neurophobia, curricular design should address the themes identified in the model. For example, to address any prior experience (Environment/Learner) that worsened neurophobia for a student, an orientation session at the beginning of a course or clerkship that includes reflection exercises might prove useful to recognize and reduce bias.

Similarly, ensuring the learning objectives for a course or clerkship are constrained to the essential (Material/Learner) might alleviate neurophobia. With characteristic enthusiasm for the field, it is easy for neurology educators to create oversized expectations for all our learners with assumptions that they will all be neurologists or with designs to recruit them (Career Perspective). To manage this without restricting students from the richness of the field, consider carefully labeling and scaffolding all the material we find to be aspirational, and clearly exclude it from high stakes examinations. Some of these principles are outlined in some recent review articles on neuroscience curriculum.^46,50^

Trying to integrate neurology learning objectives into classically non-neurology portions of the macro-curriculum might help with neurophobia (Black Box). Examples include teaching stroke during an emergency medicine rotation, headache during a family medicine rotation, and neurodegenerative disease in a geriatric medicine rotation, etc. We have started writing all our teaching cases as patients presenting in non-neurology settings (i.e., primary care clinic, ER). Longitudinal integrated clerkships (LIC), for which neurology clinical training is interwoven into continuous immersive experiences, are another great opportunity for implementation and study.

Future studies should be aimed at components of this new model to inform the creation of valid assessments and rational design of interventions with appropriate outcome measures. Assessment should be based on validated instruments of neurophobia and other appropriate outcome measures. First, we hope to validate and refine the findings with similar studies in other environments including those with different neurology curricula. We also aim to create a validated survey instrument based on this model to assess specific areas of neurophobia. Finally, we hope to study interventions like those suggested above and their effect on this model of neurophobia.

Neurophobia, the fear of, or anxiety for, the study and practice of neurology, is multidimensional with contributing factors inherent to the learner, related to the fundamental material, and influenced by the student's learning environment and prior experiences. This study offers a new model for neurophobia. The factors in the model are complex with the potential to either worsen or lessen neurophobia. Within the new model, the Black Box phenomenon, or the pervasive isolation of neurology and its adverse effects on neurophobia is highlighted. Another novel finding is the degree to which students measure their confidence in neurology as a consideration of choosing a career in neurology, and how this affects neurophobia. This new model has important implications for meaningful, sound strategies for curing neurophobia.

## Glossary

MA: math anxiety
